# Treatment Outcomes of Isoniazid-Resistant (Rifampicin Susceptible) Tuberculosis Patients in Uzbekistan, 2017–2018

**DOI:** 10.3390/ijerph18062965

**Published:** 2021-03-14

**Authors:** Zayniddin Sayfutdinov, Ajay Kumar, Dilyara Nabirova, Jamshid Gadoev, Laziz Turaev, Sanjar Sultanov, Sevak Alaverdyan, Nargiza Parpieva

**Affiliations:** 1 National Reference Laboratory, Tashkent 100086, Uzbekistan; laziz.turaev@gmail.com (L.T.); sanjarsultanov1990@gmail.com (S.S.); 2 International Union Against Tuberculosis and Lung Disease, South-East Asia Office, New Delhi 110016, India; akumar@theunion.org; 3 International Union Against Tuberculosis and Lung Disease, 75006 Paris, France; 4 Yenepoya Medical College, Yenepoya (Deemed to be University), Mangaluru 575018, India; 5 United States Centers for Disease Control and Prevention, Central Asia Region, Almaty A25T0A1, Kazakhstan; hny5@cdc.gov; 6 WHO Country Office in Uzbekistan, Tashkent 700029, Uzbekistan; gadoevj@who.int; 7 Bielefeld Graduate School of Economics and Management (BiGSEM), Bielefeld University, Universitätsstraße, 33615 Bielefeld, Germany; s.alaverdyan@iset.ge; 8 National Tuberculosis Programme, Ministry of Health, Tashkent 100086, Uzbekistan; nargizaparpieva@gmail.com

**Keywords:** Central Asia, operational research, Hr-TB, isoniazid resistance, treatment outcome, mono resistance, SORT IT (Structured Operational Research Training Initiative)

## Abstract

Tuberculosis patients “resistant to isoniazid and susceptible to rifampicin (Hr-TB)” remain neglected, despite a high burden and poor outcomes. The World Health Organization (WHO) recommends a 6 month regimen consisting of levofloxacin, rifampicin, ethambutol, and pyrazinamide (LRZE) to treat Hr-TB. In contrast, Uzbekistan uses a 9 month regimen (LRZE plus a second-line injectable in the first 3 months). We aimed to assess the treatment outcomes of this novel regimen among Hr-TB patients treated in two regions of Uzbekistan (Fergana and Bukhara) in 2017–2018. We conducted a cohort study involving secondary analysis of routine surveillance data. Of 132 Hr-TB patients, 105 (80%) were successfully treated. Death was the predominant unsuccessful outcome (13, 10%) followed by “treatment failure” (10, 8%) and “lost to follow-up” (4, 2%). High treatment success is an indicator of the potential effectiveness of the novel regimen and adds to the limited global evidence on this issue. However, the sample size was small and there was no comparison group. Since the study was conducted in two regions of Uzbekistan only, the findings have limited generalizability. We recommend future research using an adequate sample size and an appropriate study design (randomized controlled trial or prospective cohort with a control group receiving the WHO-recommended regimen).

## 1. Introduction

Tuberculosis (TB) has existed for centuries, but it remains a global threat even today. In the year 2019, about 10 million people fell ill with TB and 1.4 million people died of the disease, making TB the top cause of death among infectious diseases globally [1]. As a global community, we have pledged to end the TB epidemic by 2030 [2,3]. One of the threats preventing us from meeting the end TB targets is the epidemic of drug-resistant TB.

According to the World Health Organization (WHO), there were an estimated 465,000 people who developed TB that was resistant to rifampicin (RR-TB) in 2019, and, of these, 78% had multidrug-resistant TB (MDR-TB, defined as resistance to both isoniazid and rifampicin) [1]. The diagnosis and management of MDR/RR-TB patients have vastly improved in recent years, primarily due to widespread availability of rapid molecular diagnostics and shortened treatment regimens.

However, TB patients “resistant to isoniazid and susceptible to rifampicin” (Hr-TB) remain neglected, even though their burden seems to be higher than MDR/RR-TB. In 2019, the global burden of isoniazid-resistant TB was estimated by the WHO to be 13.1% in new TB patients and 17.4% in previously treated TB patients [1]. These proportions translate to 1.4 million patients with isoniazid-resistant TB including 1.1 million patients with Hr-TB. Since Hr-TB is not routinely diagnosed by the National TB Program (NTP), they are often treated with first-line regimens which lead to poor outcomes including high rates of treatment failure, relapse, and acquired rifampicin resistance [4]. Thus, addressing Hr-TB is crucial in order to prevent the occurrence of MDR-TB and the consequent mortality and morbidity.

Uzbekistan is one of the 30 countries in the world with a high burden of MDR/RR-TB [1]. A national drug resistance survey conducted in 2010–2011 estimated that the magnitude of “any isoniazid resistance” was high at 42% among new patients and 79% among previously treated patients, and that the burden of Hr-TB was 19% and 17% among new and previously treated patients, respectively [5].

WHO now recommends testing all TB patients for isoniazid resistance, preferably using molecular methods such as a line probe assay (LPA) and those diagnosed with Hr-TB be treated with the 6LRZE regimen (6 months of levofloxacin, rifampicin, pyrazinamide, and ethambutol) [6,7]. It is also recommended that streptomycin or any other injectable should not be used as part of this regimen. However, this is a conditional recommendation with very low certainty, because there is no evidence about the optimal regimen for treating Hr-TB from randomized controlled trials (RCTs) [7].

To diagnose Hr-TB, the Uzbekistan NTP started offering first-line LPA in 2015 on a pilot basis (Ministry of Health Order #383), which became more widely available in 2017. Patients diagnosed with Hr-TB have been treated using a 9 month regimen that includes levofloxacin throughout the treatment with injectable second-line drugs included in the first 3 months. This is a different regimen than that recommended by the WHO, and there has not been any systematic assessment up to now as to how well this regimen is performing in terms of treatment outcomes. Unfortunately, disaggregated data about outcomes of Hr-TB patients are not routinely compiled and reported. Furthermore, there is no published evidence on this issue from Uzbekistan. Hence, we conducted this operational research study to assess the treatment outcomes and factors associated with unsuccessful treatment outcomes among patients with Hr-TB treated in two regions of Uzbekistan in 2017 and 2018.

## 2. Materials and Methods

### 2.1. Study Design

This was a cohort study involving secondary analysis of routine data collected by the NTP in Uzbekistan.

### 2.2. Setting

Uzbekistan is a country in Central Asia with a population of 33 million people. Uzbekistan consists of 12 regions (or oblasts), one autonomous republic (the Republic of Karakalpakstan), and the Tashkent metropolitan area, the capital city.

#### 2.2.1. TB Control Program in Uzbekistan

TB control activities are coordinated countrywide by the NTP based out of the Republican Specialized Scientific Practical Medical Center of Phthisiology and Pulmonology. TB diagnosis and treatment are provided free of charge within the NTP. There are no private TB services. All the drugs used in the NTP are quality-assured and procured through the support of the Global Fund and the domestic budget.

#### 2.2.2. Diagnosis of Hr-TB

Sputum samples of TB patients suspected of isoniazid resistance by the treating clinicians are collected and transported to one of the regional or national laboratories for testing. Hr-TB is diagnosed using first-line LPA [GenoType MTBDRplus VER 1 and 2, Hain Lifescience, Germany] at the following laboratories: two national reference laboratories (NRLs)—one in Tashkent City and another in Nukus, Karakalpakstan—and five inter-regional laboratories (Bukhara, Fergana, Surkhandarya, Samarkand, and Tashkent regions). NRLs are monitored by the Supra National Reference Laboratories in Borstel and Gauting, Germany.

#### 2.2.3. Treatment of Hr-TB

While awaiting results of isoniazid resistance, patients are treated with first-line regimens as appropriate. Once diagnosed as Hr-TB (irrespective of drug susceptibility pattern to other drugs such as ethambutol, pyrazinamide, and streptomycin), patients are switched to a 9 month regimen. This consists of 3 months of the intensive phase (IP) and 6 months of the continuation phase (CP). During the IP, five drugs are given, which include kanamycin or capreomycin and LRZE. During the continuation phase, four drugs are given (LRZE).

Uzbekistan has a predominantly hospital-based health system. All TB patients are admitted at the regional TB hospitals or district-level TB dispensaries for the entire duration of the intensive phase. Once the intensive phase is completed or once the patient becomes negative for sputum smear or culture (whichever is earlier), the patient is discharged and referred to the nearest primary health center (PHC) for continuation of treatment on an outpatient basis. At the PHC, patients are expected to visit daily and receive the treatment under the direct observation of a healthcare provider. If the patient is very sick, a nurse from the PHC visits the patient at home and provides the treatment.

All patients are routinely tested for human immunodeficiency virus (HIV) and those found HIV-positive are provided antiretroviral therapy (ART) and cotrimoxazole preventive therapy (CPT), irrespective of CD4 (cluster of differentiation 4) count or clinical stage. Treatment outcomes are standardized and follow the WHO guidelines.

#### 2.2.4. Recording and Reporting

The details about Hr-TB diagnosis are captured in the laboratory database maintained at the NRLs and the inter-regional laboratories. The details of treatment are captured on the “treatment card” and the “TB Register”. A treatment card is opened for every new patient started on treatment at the TB dispensary or the hospital. At the time of discharge, the treatment card is sent with the patient to the referred PHC, and a copy is maintained at the TB dispensary/hospital. At the end of the treatment, the treatment card is sent back from the PHC to the TB dispensary/hospital for permanent archiving. The key patient details from the treatment card are abstracted into the TB register maintained at the TB dispensary/hospital.

### 2.3. Study Population

All Hr-TB patients diagnosed and treated in two regions (Bukhara and Fergana) of Uzbekistan, from January 2017 to December 2018, were included.

### 2.4. Data Variables and Sources

The key variables included age, sex, region, type of TB, culture results (sourced from laboratory database at the NRL), previous history of TB treatment, HIV status, and treatment outcome (sourced from TB registers maintained at the district-level TB dispensaries/hospitals).

### 2.5. Analysis and Statistics

Data were analyzed using R software (version 4.0.3). Demographic, clinical, and treatment characteristics were summarized as percentages. TB treatment outcomes were categorized into successful (cured and treatment completed) and unsuccessful (failure, lost to follow up, death, or not evaluated) outcomes. The chi-square test or Fischer’s exact test was used to test if the difference between proportions was statistically significant. A *p*-value < 0.05 was considered statistically significant. Unadjusted relative risks (RR) with 95% confidence intervals (CI) were calculated to assess associations. Given the small sample size and missing data in several exposure variables, we considered it prudent not to conduct a multivariable analysis.

## 3. Results

### 3.1. Baseline Characteristics

There were a total of 132 Hr-TB patients in our study sample (Table 1). Of these, 59 (45%) were female and only five (4%) were children aged <18 years. Most (94%) of the patients had pulmonary TB and nearly half (41%) were culture-positive for *Mycobacterium tuberculosis*. All the patients were tested for HIV, and only one person was HIV-positive, who was started on ART.

### 3.2. Treatment Outcomes

Of 132 patients, 105 (80%) were successfully treated (Table 2). Death was the predominant unsuccessful outcome and was seen in 13 (10%) of the patients, followed by “treatment failure” in 10 (8%) patients. None of the factors studied were significantly associated with unsuccessful outcomes (Table 3).

## 4. Discussion

This is the first study from Uzbekistan assessing treatment outcomes among Hr-TB patients. Treatment outcomes were good with nearly 80% of the patients successfully completing the treatment. While we do not know the exact reasons for this observation, this may be an indirect indicator of the effectiveness of the treatment regimen used. Globally, there is limited evidence about the effectiveness of treatment regimens used for treating Hr-TB [6,7]. In contrast to the WHO-recommended 6 month therapy (LRZE), Uzbekistan is using a standardized 9 month treatment regimen for Hr-TB patients that includes levofloxacin throughout the treatment with injectable second-line drugs included in the first 3 months. This is the first global experience of using such a new treatment regimen.

Death was the predominant unsuccessful outcome accounting for nearly 10% of all patients, followed by treatment failure in 8% of patients. This may be due to the fact that all the Hr-TB patients were given the same regimen irrespective of the resistance pattern. It is possible that many patients had additional resistance to ethambutol and pyrazinamide which might have rendered the standard regimen ineffective. Since we did not have the results of drug susceptibility to ethambutol and pyrazinamide, we are unable to comment further on this issue.

There were a number of limitations in this study. First, TB patients were not routinely tested for isoniazid resistance during the study period. Only those patients who were referred by clinicians were tested. It is unclear what criteria were used to refer patients for testing, but we speculate that it was done for patients not responsive to first-line treatment. Hence, we cannot rule out a selection bias, which might partially explain the poor outcomes. Second, the data came from two regions only and, hence, are not representative of the situation in Uzbekistan. Third, the sample size was small, and this precluded a robust analysis of factors associated with poor outcomes. Fourth, we had no information on adverse drug events. This is important given the use of injectables in the first 3 months which are notorious for causing ototoxicity. We feel it was a missed opportunity to assess the frequency of ototoxicity, which may be progressive and permanent and can severely affect quality of life. Fifth, data on some key variables such as the DST pattern were missing, which might have helped in explaining the reasons for poor outcomes. Lastly, we did not have an explicit comparison group within our study. A comparison group receiving the WHO-recommended regimen would have enabled us to make head-to-head comparison in the study. This was not possible because all the Hr-TB patients in Uzbekistan receive the 9 month regimen. We were also unable to constitute a historical control group because the isoniazid drug susceptibility testing was not widely available in previous years and the isoniazid resistance status was not routinely recorded on treatment cards.

Despite these limitations, we feel that the novel treatment regimen used for treatment of Hr-TB in Uzbekistan holds promise given the high treatment success observed in this study. We recommend that future research be conducted using adequate sample size and an appropriate study design addressing the limitations of the current study. Ideally, an RCT is required to identify the most efficacious treatment regimen for Hr-TB. However, RCTs are resource-intensive and may take a long period of time to conduct. As the next best alternative, we recommend a prospective cohort study (with an appropriate comparison group receiving the WHO-recommended regimen).

In our view, Hr-TB has not received the attention and priority it deserves over the years. The WHO routinely requests from countries treatment outcome information for four groups of patients: (i) patients treated with first line regimens, (ii) HIV-associated TB patients, (iii) MDR/RR-TB patients, and (iv) extensively drug-resistant TB (XDR-TB) patients. This is compiled and reported every year in the global TB reports published by the WHO. However, treatment outcomes have never been segregated for Hr-TB patients, and countries do not analyze and review the data of Hr-TB patients routinely. This needs to change given that the burden of Hr-TB far exceeds that of MDR/RR-TB patients and patients can have poor outcomes, if inadequately treated.

## 5. Conclusions

In conclusion, high treatment success was achieved among Hr-TB patients in Uzbekistan using a novel, 9 month standardized regimen containing levofloxacin throughout the treatment. This study adds to the limited global evidence on this issue. However, the sample size was small and there was no comparison group. Since the study was conducted in two regions of Uzbekistan only, the findings have limited generalizability. We hope that these findings will stimulate future research addressing the limitations of the current study.

## Figures and Tables

**Table 1:** Demographic and clinical characteristics of TB patients “resistant to isoniazid and susceptible to rifampicin” (Hr-TB) treated in two regions of Uzbekistan from January 2017 to December 2018.

Variable	*N* (%)
**Total**	132 (100)
**Sex**	
Male	73 (55.3)
Female	59 (44.7)
**Age (years)**	
<18	5 (3.8)
18–44	24 (18.2)
45–59	41 (31.1)
60–74	33 (25.0)
75–90	29 (22.0)
**Region**	
Fergana	57 (43.2)
Bukhara	75 (56.8)
**TB type**	
Pulmonary	124 (93.9)
Extrapulmonary	8 (6.1)
**Previous treatment history**	
New	43 (32.6)
Previously treated	89 (67.4)
**HIV status**	
Positive	1 (0.8)
Negative	131 (99.2)
**Cavitary lesion**	
Yes	12 (9.1)
No	120 (90.9)
**TB culture**	
Positive	54 (40.9)
Negative	69 (52.3)
Missing	9 (6.8)
**Sputum microscopy**	
Positive	59 (44.7)
Negative	18 (13.6)
Missing	55 (41.7)

TB = tuberculosis; HIV = human immunodeficiency virus; H = isoniazid; S = streptomycin; Hr-TB = isoniazid-resistant (rifampicin-susceptible) TB.

**Table 2:** Treatment outcomes of isoniazid-resistant (rifampicin-susceptible) TB patients treated in two regions of Uzbekistan from January 2017 to December 2018.

Treatment Outcome	*N*	(%)
**Total**	132	(100)
**Successful**	105	(79.5)
Cured	94	(71.2)
Treatment completed	11	(8.3)
**Unsuccessful**	27	(20.5)
Died	13	(9.8)
Failure	10	(7.6)
Lost to follow-up	4	(3.1)

**Table 3:** Factors associated with unsuccessful outcomes among Hr-TB patients treated in two regions of Uzbekistan from January 2017 to December 2018.

Variable	Total	Unsuccessful		RR	95% CI	*p*-Value
	N	N	(%)			
**Total**	132	27	(20.5)			
**Sex**						
Male	73	18	(24.7)	1.62	[0.78, 3.33]	0.183
Female	59	9	(15.3)	Ref.		
**Age (years)**						
<18	5	1	(20.0)	1.32	[0.19, 9.09]	0.782
18–44	24	5	(20.8)	1.38	[0.45, 4.22]	0.578
45–59	41	8	(19.5)	1.29	[0.46, 3.57]	0.624
60–74	33	5	(15.2)	Ref.		
75–90	29	8	(27.6)	1.82	[0.67, 4.95]	0.230
**Region**						
Fergana	57	13	(22.8)	1.22	[0.62, 2.39]	0.559
Bukhara	75	14	(18.7)	Ref.		
**TB type**						
Pulmonary	124	26	(21.0)	1.68	[0.26, 10.83]	0.565
Extrapulmonary	8	1	(12.5)	Ref.		
**Previous treatment history**						
New case	43	10	(23.3)	1.22	[0.61, 2.43]	0.579
Previously treated	89	17	(19.1)	Ref.		
**HIV status**						
Positive	1	0	(0.0)	Inf.	[NA, Inf.]	0.611
Negative	131	27	(20.6)	Ref.		
**Cavitary lesion**						
Yes	12	3	(25.0)	1.25	[0.44, 3.55]	0.682
No	120	24	(20.0)	Ref.		
**TB culture**						
Positive	54	13	(24.1)	1.19	[0.61, 2.31]	0.615
Negative	69	14	(20.3)	Ref.		
Missing	9	0	(0.0)	0	[0, NA]	0.136
**Sputum microscopy**						
Positive	59	11	(18.6)	3.36	[0.46, 24.26]	0.180
Negative	18	1	(5.6)	Ref.		
Missing	55	15	(27.3)	4.91	[0.70, 34.61]	0.053

TB = tuberculosis; HIV = human immunodeficiency virus; Hr-TB = isoniazid-resistant (rifampicin-susceptible) TB; Ref. = reference group; NA = not applicable; Inf = infinity.

## Data Availability

The data that support the findings of this study are available from the corresponding author, Z.S., upon reasonable request.

## Disclaimer

The authors alone are responsible for the views expressed in this publication and they do not necessarily represent the decisions or policies of the World Health Organization. ©World Health Organization 2021. All rights reserved. The WHO Regional Office for Europe has granted the Publisher permission for the reproduction of this article.

## Open Access Statement

In accordance with WHO’s open-access publication policy for all work funded by WHO or authored/co-authored by WHO staff members, the WHO retains the copyright of this publication through a Creative Commons Attribution IGO license (http://creativecommons.org/licenses/by/3.0/igo/legalcode) which permits unrestricted use, distribution, and reproduction in any medium provided the original work is properly cited.
